# Supplementary material for: Reduction of Lams-Related Adverse Events with Accumulating Experience in a Large-Volume Tertiary Referral Center
Source: J Clin Med. 2023 Jan 29;12(3):1037. doi: 10.3390/jcm12031037 (PMC9917779; doi:10.3390/jcm12031037)
Supplement: Supplementary file 1 [file jcm-12-01037-s001.zip › jcm-2150032-supplementary.pdf]

Supplementary Table S1. Comparison with the previously published cohort.[6]

	Number of procedures	Technical success	Clinical success	Adverse events
**PFC current**	40/168 (23.8%)	38/40 (95.0%)	35/40 (87.5%)	2/40 (5.0%)
**PFC previous**	24/61 (39.3%)	22/24 (91.7%)	20/24 (83.3%)	8/24 (33.3%)
**GE current**	35/168 (20.8%)	33/35 (94.3%)	33/35 (94.3%)	3/35 (8.6%)
**GE previous**	6/61 (9.8%)	6/6 (100.0%)	6/6 (100.0%)	0/6 (0.0%)
**EUS – BD current**	21/168 (12.5%)	21/21 (100.0%)	20/21 (95.2%)	2/21 (9.5%)
**EUS – BD previous**	13/61 (21.3%)	12/13 (92.3%)	11/13 (84.6%)	3/13 (23.1%)
**EUS – GBD current**	27/168 (16.1%)	27/27 (100.0%)	27/27 (100.0%)	5/27 (18.5%)
**EUS – GBD previous**	1/61 (1.6%)	1/1 (100.0%)	1/1 (100.0%)	0/1 (0.0%)
**GATE – current**	25/168 (14.8%)	25/25 (100.0%)	25/25 (100.0%)	1/25 (4.0%)
**GATE - previous**	13/61 (21.3%)	13/13 (100.0%)	13/13 (100.0%)	1/13 (7.7%)
**Treatment of refractory GI strictures – current**	6/168 (3.6%)	6/6 (100.0%)	4/6 (66.7%)	0/6 (0.0%)
**Treatment of refractory GI strictures - previous**	NA	NA	NA	NA
**Misscelaneous – current**	12/168 (7.1%)	11/12 (91.7%)	11/12 (91.7%)	3/12 (25.0%)
**Misscelaneous - previous**	4/61 (6.6%)	3/4 (75.0%)	3/4 (75.0%)	1/4 (25.0%)

PFC – peripancreatic fluid collections, GE – gastroenterostomy, EUS BD – endoscopic ultrasound guided biliary drainage, EUS GBD – endoscopic ultrasound guided gallbladder drainage, GATE – temporary access for endoscopic procedures, GI – gastrointestinal. Misscelaneous indications in the current cohort are discussed in the manuscript, however in the previously published cohort, misscelaneous meant only postsurgical collections.
